# Comparison of the Fully Automated FilmArray BCID Assay to a 4-Hour Culture Test Coupled to Mass Spectrometry for Day 0 Identification of Microorganisms in Positive Blood Cultures

**DOI:** 10.1155/2018/7013470

**Published:** 2018-11-21

**Authors:** Paul O. Verhoeven, Cyrille H. Haddar, Josselin Rigaill, Nathalie Fonsale, Anne Carricajo, Florence Grattard, Bruno Pozzetto

**Affiliations:** ^1^GIMAP EA 3064 (Groupe Immunité des Muqueuses et Agents Pathogènes), University of Lyon, Faculty of Medicine of Saint-Etienne, Saint-Etienne, France; ^2^Laboratory of Infectious Agents and Hygiene, University Hospital of Saint-Etienne, Saint-Etienne, France

## Abstract

Rapid bacterial identification of positive blood culture is important for adapting the antimicrobial therapy in patients with blood stream infection. The aim of this study was to evaluate the performance of the multiplex FilmArray Blood Culture Identification (BCID) assay by comparison to an in-house protocol based on MALDI-TOF MS identification of microcolonies after a 4-hour culture, for identifying on the same day the microorganisms present in positive blood culture bottles. One hundred and fifty-three positive bottles from 123 patients were tested prospectively by the 3 techniques of bacterial identification: 11 bottles yielding negative results by the 3 tests were considered false positive (7.2%). The reference MALDI-TOF MS technique identified 134 monomicrobial (87.6%) and 8 double infections (5.2%), which resulted in a total of 150 microorganisms. Globally, 137 (91.3%) of these 150 pathogens were correctly identified by the fully automated multiplex FilmArray BCID system at the species or genus level on day of growth detection, versus 117 (78.8%) by MALDI-TOF MS identification on nascent microcolonies after a 4-hour culture (*P *< 0.01). By combining the two approaches, 140 (93.5%) of the positive bottles were identified successfully at day 0. These results confirm the excellent sensitivity of the FilmArray BCID assay, notably in case of multimicrobial infection. Due to the limited number of targets included into the test, it must be coupled to another identification strategy, as that presented in this study relying on MALDI-TOF MS identification of microcolonies obtained after a very short culture period.

## 1. Introduction

Rapid bacterial identification of positive blood culture is important for adapting the antimicrobial therapy in patients with blood stream infection [1]. Matrix assisted laser desorption ionization-time of flight mass spectrometry (MALDI-TOF MS) has considerably shortened the time for identifying bacteria recovered from positive blood cultures [2]. In order to perform the identification of pathogens on the same day after detection of growth, different strategies have been proposed relying on lysis/filtration method coupled to MALDI-TOF MS [3–8], on rapid identification by MALDI-TOF MS of nascent colonies obtained after plating blood cultures for a short-incubation period [9–11], or on direct identification of pathogens present in blood cultures using fully automated PCR assays [12–21].

The aim of this study was to evaluate the performance of the multiplex FilmArray Blood Culture Identification (BCID) assay by comparison to an in-house protocol based on the MALDI-TOF MS identification after a 4-hour culture (termed “fast MALDI-TOF MS”) [9] for identifying on the same day the microorganisms present in positive blood culture bottles.

## 2. Material and Methods

### 2.1. Design of the Study

Blood cultures detected positive during the laboratory operating hours by the BD BACTEC 9240 system (Plus Aerobic/F and Plus Anaerobic/F bottles) (Becton Dickinson, Meylan, France) in patients hospitalized at the University Hospital of Saint-Etienne on a period of 10 months were included into the study with the limitation of a single aerobic or anaerobic vial per puncture site, per patient, and per day to increase the range of microorganisms responsible for blood stream infection.

### 2.2. Techniques

For the reference technique, medium from positive bottles was gram-stained and inoculated onto agar plates with incubation under aerobic and anaerobic atmosphere until a growth was observed (average time of 21 hours). Resulting colonies were identified by MALDI-TOF MS (Microflex LT, Bruker Daltonik GmbH, Bremen, Germany) using MALDI Biotyper v3.0 software. MALDI Biotyper scores greater than or equal to 1.7 and 1.9 were used for identification at genus and species levels, respectively, according to the thresholds previously described [22].

**Figure 1:**
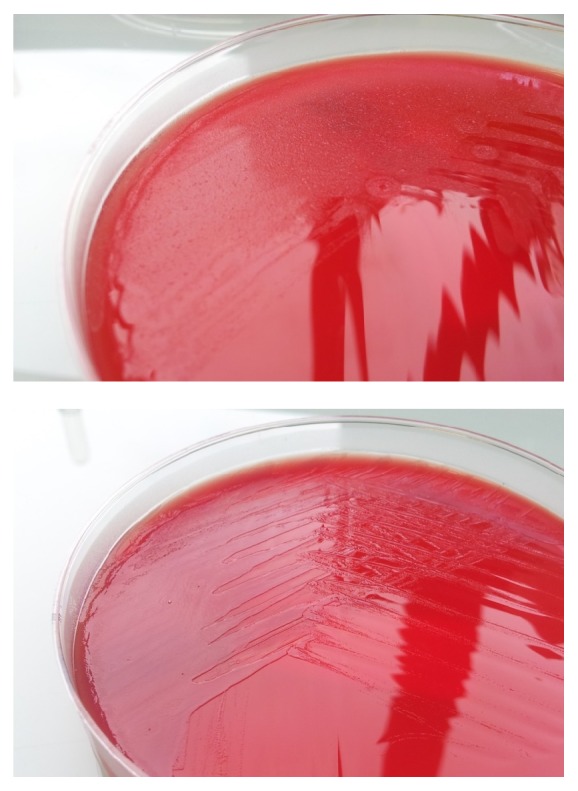
Pictures of microcolonies of* Staphylococcus aureus* (upper panel) and* Escherichia coli* (lower panel) on blood agar after four hours of incubation at 37°C under 5% of CO_2_.

For the fast MALDI-TOF MS assay, microcolonies recovered from agar plates after 4 hours of incubation under appropriate conditions (Figure 1) were recovered and submitted to MALDI-TOF MS analysis. MALDI Biotyper scores greater than or equal to 1.5 were used for bacterial identification; this threshold was deduced from a previous study that we performed to validate the use of MALDI-TOF MS for bacterial identification from positive blood cultures after short-time culture [9].

The FilmArray BCID assay (BioFire Diagnostics, Salt Lake City, UT, USA, and bioMérieux, Marcy l'Etoile, France) was performed following manufacturer's recommendations. Briefly, a 200 *µ*l-volume of positive blood culture bottle was added to the red buffer and vortexed shortly. Then, blue and red buffers were transferred into the cartridge using the provided syringes. Extraction, amplification, and reading steps were fully automated into the Biofire instrument. Results that were analyzed by the software were available within 1 hour of time through a report indicating the detected microorganism(s).

### 2.3. Statistical Analyses

Descriptive variables were reported with their 95% confidence interval (CI). The Chi-square test was used for the comparison of qualitative variables; the two-tailed Fisher exact test was preferred in case of small effectives.* P* values under 5% were considered to be statistically significant.

## 3. Results

One hundred and fifty-three (153) positive blood culture bottles from 123 patients were tested prospectively by the 3 techniques of bacterial identification. Eleven bottles yielded negative results by the 3 tests and were considered false positive (7.2%). The reference MALDI-TOF MS technique identified 134 monomicrobial (87.6%) and 8 double infections (5.2%), which resulted in the identification of a total of 150 microorganisms listed in Table 1.

The fast MALDI-TOF MS technique missed 33 microorganisms that gave either no significant growth in 4 hours (10 cases) or a score under 1.5 (13 cases), including 6 of the 11* Enterococcus *spp. and the 3 yeasts (Table 1). Concerning the 8 double infections, 10 of 16 germs were missed (62.5%): 1 germ in 6 cases and 2 germs in 2 cases (Table 2).

The FilmArray BCID test missed 13 microorganisms, including 10 agents that were off-panel (Table 1), one strain of* S. warneri* (this species was mentioned as ill-recognized in the booklet of the test), one strain of* S. gallolyticus*, and one strain of* S. aureus* (identified as* Staphylococcus* spp.). Extraction control or amplification control included in the cartridge was not amplified in 3 cases (2.0%) but a correct identification was obtained after the second run. In 6 bottles, an additional germ was identified by comparison to the reference technique (2 strains of* Staphylococcus* spp., 1 strain of* S. aureus*, 1 strain of* Streptococcus* spp., 1 strain of* E. coli*, and 1 strain of* K. pneumoniae*). The 8 coinfections detected by the reference technique were also detected by the FilmArray BCID assay; by comparison to the reference technique, an additional strain of* Staphylococcus *spp. was identified in one bottle whereas a strain of* Streptococcus* spp. was missed in another bottle (Table 2).

Concerning antimicrobial resistances detected by FilmArray BCID, no isolate was found to harbor* van*A/*van*B or KPC genes. Four of the 22 isolates of* S. aureus* (18.2%) and 35 of the 41 coagulase negative staphylococci (85.4%) were detected positive for the* mec*A gene by FilmArray BCID. Resistance to oxacillin was confirmed by standard antimicrobial susceptibility testing for all isolates (MIC greater than or equal to 4 mg/l) but 5 coagulase negative staphylococci that were not tested.

Table 3 depicts the performances of the two rapid tests by comparison to the reference method for the 153 positive blood specimen bottles. The global agreement of the FilmArray BCID with the reference technique was 91.5% [95% CI: 87.1-95.9], significantly higher than that with the fast MALDI-TOF MS assay (79.7% [95% CI: 73.4-86.0],* P* < 0.01 by Chi-square test). The sensitivity of the FilmArray BCID assay was 90.9% [95% CI: 86.1-95.6], significantly higher than that of the fast MALDI-TOF method (78.2% [95% CI: 71.4-85.0],* P* < 0.01 by Chi-square test). By combining the two approaches, 93.5% of the bottles were identified correctly at day 0.

## 4. Discussion

These results confirm the excellent sensitivity of the FilmArray BCID assay reported in previous studies [13, 14, 20, 21, 23–27], notably in case of multimicrobial infection. Although no sequencing was performed in this study, it was previously shown that microorganisms identified only by FilmArray BCID could be detected by sequencing, showing that they were true positives [13]. By contrast to other rapid techniques coupled to MALDI-TOF MS, the hands-on time is only of 3-5 minutes and the test does not require trained personnel. The ability to detect resistance genes to some antimicrobial agents is a further advantage.

Due to the limited number of targets included into the test, it must be coupled to another identification strategy. Its high cost and the need to test each sample individually may represent further limitation in laboratories with high test volume [14]. Although less sensitive, notably for slow-growing microorganisms, the 4-hour incubation MALDI-TOF MS method used as comparator in this study represents a much cheaper alternative.

As suggested by Fiori et al. [28], it could be interesting to reserve the FilmArray BCID approach to those bottles that failed identification by the fast MALDI-TOF MS method (≈20% of the bottles tested in this study). The study of Pardo et al. [29] demonstrated that the FilmArray BCID assay, when coupled with antimicrobial stewardship intervention, was a cost-effective tool to improve patient care. In a recent meta-analysis including 31 studies and 5920 patients, Timbrook et al. [30] showed that molecular rapid diagnostic tests in bloodstream infections were associated with significant decrease in mortality risk if associated with an antimicrobial stewardship program. Similar conclusions were drawn from previous reviews on the same topic [31, 32]. Consequently, it could be interesting to target this identification approach to patients considered as the more critical and for whom the rapid adaptation of the antimicrobial therapy represents a key issue in terms of prognosis.

## 5. Conclusions 

This study confirms the excellent analytical performances of the FilmArray BCID assay for microorganism identification at day 0 in positive blood cultures, notably in case of polymicrobial infections (including yeasts). When combined with a 4-hour culture test coupled to mass spectrometry, it was able to give a correct result in more than 93% of the tested cases. Due to high cost and limited targets, the FilmArray BCID could be dedicated to the more severe patients with suspected sepsis who need a quick adjustment of their antimicrobial treatment. In these patients, the analysis of the literature indicates that the implementation of the test must be combined with an antimicrobial stewardship program to provide significant clinical results.

## Figures and Tables

**Table 1:** Number of pathogens detected in the 153 blood specimen bottles by the three techniques that were compared in this study. The MALDI-TOF MS technique was taken as the gold standard.

	Reference technique (MALDI-TOF MS)	MALDI-TOF MS at day 0 (% according to reference)	FilmArray BCID assay (% according to reference)	*P *value^1^
**All pathogens**	**150**	**117 (78.0)**	**137 (91.3)**	**< 0.01**
Gram positive bacteria detected by the FilmArray BCID panel	85	70 (82.4)	82 (96.5)	< 0.01
(i) *Staphylococcus aureus*	23	22 (95.7)	22 (95.7)^2^	
(ii) *Coagulase negative staphylococci*^*3*^	42	36 (85.7)	41 (97.6)^4^	
(iii) *Streptococcus pneumoniae*	3	2 (66.7)	3 (100)	
(iv) *Streptococcus agalactiae*	0	0	0	
(v) *Streptococcus pyogenes*	0	0	0	
(vi) *Streptococcus *spp.^*5*^	5	4 (80.0)	4 (80.0)	
(vii) *Enterococcus *spp.^*6*^	11	5 (45.5)	11 (100)	
(viii) *Listeria monocytogenes*	1	1 (100)	1 (100)	
Gram negative bacteria detected by the FilmArray BCID panel	52	45 (86.5)	52 (100)	<0.05
(i) *Acinetobacter baumannii*	0	0	0	
(ii) *Haemophilus influenzae*	1	0	1 (100)	
(iii) *Neisseria meningitidis*	0	0	0	
(iv) *Pseudomonas aeruginosa*	0	0	0	
(v) *Enterobacteriaceae*	51	45 (88.2)	51 (100)	
(a) *Enterobacter cloacae complex*	3	3 (100)	3 (100)	
(b) *Escherichia coli*	34	31 (91.2)	34 (100)	
(c) *Klebsiella oxytoca*	2	0	2 (100)	
(d) *Klebsiella pneumoniae*	9	8 (88.9)	9 (100)	
(e) *Proteus *spp.	1	1 (100)	1 (100)	
(f) *Serratia marcescens*	2	2 (100)	2 (100)	
Yeasts detected by the FilmArray BCID panel	3	0	3 (100)	NS
(i) *Candida albicans*	2	0	2 (100)	
(ii) *Candida glabrata*	0	0	0	
(iii) *Candida krusei*	1	0	1 (100)	
(iv) *Candida parapsilosis*	0	0	0	
(v) *Candida tropicalis*	0	0	0	
Microorganisms absent from the FilmArray BCID panel but detected by MALDI-TOF MS	10	2 (20.0)	0	NS
(i) *Gemella haemolysans*	1	0	0	
(ii) *Gemella *spp.	1	0	0	
(iii) *Corynebacterium amycolatum*	2	0	0	
(iv) *Acinetobacter ursingii*	1	0	0	
(v) *Clostridium perfringens*	1	0	0	
(vi) *Clostridium ramosum*	1	1 (100)	0	
(vii) *Bacteroides ovatum*	1	1 (100)	0	
(viii) *Propionibacterium *spp.	1	0	0	
(ix) *Fusarium *spp.	1	0	0	

^1^The two-tailed Fisher exact test was used. NS: not significant at the level of 5%.

^2^Four strains of *S. aureus* were positive for the *mec*A gene.

^3^The 42 strains of coagulase negative staphylococci included 35 strains of *S. epidermidis*, 3 strains of *S. capitis*, 2 strains of *S. hominis*, and 1 strain of *S. caprae*, *S. haemolyticus*, and *S. warneri*, each.

^4^Thirty-five strains of coagulase negative staphylococci were positive for the *mec*A gene.

^5^The 5 strains of *Streptococcus* spp. included 2 strains of *S. gallolyticus*, 2 strains of *S. mitis/oralis*, and 1 strain of *S. parasanguinis*.

^6^The 11 strains of *Enterococcus* spp. included 6 strains of *E. faecalis*, 3 strains of *E. faecium*, 1 strain of *E. gallinarum*, and 1 strain of *E. hirae*.

**Table 2:** Pathogens identified in the 8 blood culture bottles exhibiting coinfection.

Reference technique (MALDI-TOF MS)	MALDI-TOF MS at day 0	FilmArray BCID assay
*E. coli / K. oxytoca*	*K. oxytoca*	*E. coli / K. oxytoca*
*E. coli / K. oxytoca*	*K. oxytoca*	*E. coli / K. oxytoca*
*E. coli / K. pneumoniae*	*E. coli*	*E. coli / K. pneumoniae*
*E. coli / K. pneumoniae*	*E. coli*	*E. coli / K. pneumoniae*
*E. coli / E. faecalis*	*E. faecalis*	*E. coli / E. faecalis*
*E. coli / E. hirae*	-	*E. coli / E. hirae / Streptococcus* spp.
*S. epidermidis / S. hominis*	*S. hominis*	*Staphylococcus* spp. (+ *mec*A gene)
*C. albicans / C. krusei*	-	*C. albicans / C. krusei*

**Table 3:** Comparative results of the two rapid techniques on the 153 blood culture bottles of the study. Success was defined by the capacity to obtain the same result (absence or presence of one or two microorganisms) as that of the reference method (MALDI-TOF MS).

		MALDI-TOF MS at day 0 after a 4-hour culture	
		success	failure
FilmArray BCID assay	success	119	21
	failure	3	10

## Data Availability

The data used to support the findings of this study are available from the corresponding author upon request.
